# Exploring Acetogenesis in Firmicutes: From Phylogenetic Analysis to Solid Medium Cultivation with Solid-Phase Electrochemical Isolation Equipments

**DOI:** 10.3390/microorganisms11122976

**Published:** 2023-12-13

**Authors:** Zen-ichiro Kimura, Hiroki Kuriyama, Yuki Iwasaki

**Affiliations:** Department of Civil and Environmental Engineering, National Institute of Technology, Kure College, 2-2-11 Aga-minami, Kure, Hiroshima 737-8506, Japan; hiroki070621@gmail.com (H.K.); y-iwasaki@kure.kosen-ac.jp (Y.I.)

**Keywords:** microbial electrosynthesis, isolation systems, electrotrophic acetogen

## Abstract

This study introduces a groundbreaking approach for the exploration and utilization of electrotrophic acetogens, essential for advancing microbial electrosynthesis systems (MES). Our initial focus was the development of Solid-Phase Electrochemical Isolation Equipment (SPECIEs), a novel cultivation method for isolating electrotrophic acetogens directly from environmental samples on a solid medium. SPECIEs uses electrotrophy as a selection pressure, successfully overcoming the traditional cultivation method limitations and enabling the cultivation of diverse microbial communities with enhanced specificity towards acetogens. Following the establishment of SPECIEs, we conducted a genome-based phylogenetic analysis using the Genome Taxonomy Database (GTDB) to identify potential electrotrophic acetogens within the Firmicutes phylum and its related lineages. Subsequently, we validated the electrotrophic capabilities of selected strains under electrode-oxidizing conditions in a liquid medium. This sequential approach, integrating innovative cultivation techniques with detailed phylogenetic analysis, paves the way for further advances in microbial cultivation and the identification of new biocatalysts for sustainable energy applications.

## 1. Introduction

In recent years, escalating environmental concerns and the depletion of traditional energy resources have underscored the need for sustainable energy production and more efficient carbon cycling methods [1,2,3]. In this context, microorganisms, particularly electrotrophic acetogens, have emerged as being critical to ecosystem balance and carbon cycling [4]. These bacteria, capable of using extracellular electrons to reduce carbon dioxide into acetate, present a promising avenue for research due to their potential contributions to carbon fixation and sustainable energy production [5,6].

Electrotrophic acetogens are characterized by their use of the Wood–Ljungdahl pathway (WLP), a unique metabolic process for CO_2_ fixation into acetate [7]. This group of microorganisms is diverse, with some members exhibiting the capability to harness extracellular electron sources, such as cathodes, for CO_2_ reduction [8]. This distinct ability positions electrotrophic acetogens as prime candidates for various biotechnological applications, including microbial electrosynthesis (MES), bioremediation, and sustainable energy production [9].

MES represents a cutting-edge technological advancement, utilizing electrotrophic microorganisms as biocatalysts to transform CO_2_ and electrical energy into valuable organic compounds like biofuels and chemicals [10]. Beyond mere substance production, MES has been proposed for more value-added applications, such as coupling with biogas modification and biogas desulfurization processes, thereby amplifying its potential and foundational importance [11,12]. Electrotrophic acetogens, pivotal to MES, facilitate the sustainable production of value-added chemicals and fuels through their extracellular electron-utilizing capacity for CO_2_ reduction [13].

Typically, electrotrophic acetogens produce acetate from CO_2_ via WLP, which involves ATP synthesis during the conversion of acetyl phosphate into acetate [14]. However, they also possess metabolic pathways that can utilize excess electrons to produce other substances, with ethanol being the most readily inducible electron sink [15]. This versatility suggests that MES could extend its applications beyond acetate production to include fuels and chemical production, such as ethanol. Understanding the diversity, metabolic mechanisms, and ecological roles of electrotrophic acetogens is, thus, vital for the development and optimization of MES [16].

Previous studies, focusing on known electrotrophic acetogens like *Sporomusa ovata* and *Clostridium ljungdahlii*, have shed light on their metabolic mechanisms and physiological traits [17,18]. These acetogens’ ability to use cathodes and other electron donors for CO_2_ reduction is well-documented, yet the broader spectrum of electrotrophic acetogens remains largely unexplored [18]. The critical aspect of how these bacteria internalize electrons—a pivotal step for their metabolism—is still not fully understood. This gap in knowledge presents a significant hurdle in optimizing MES [19]. In response to these challenges, our study introduces SPECIEs (Solid-Phase Electrochemical Isolation Equipment), an innovative method aimed at effectively cultivating electrotrophic acetogens directly from environmental samples on a solid medium.

The unique attribute of SPECIEs is its utilization of electrotrophy as a selective force, potentially preserving the original microbial diversity while isolating specific electrotrophic organisms. This novel method addresses the shortcomings of conventional cultivation techniques, which frequently fail to directly culture these bacteria from environmental samples, thus paving the way for advanced research and applications in MES [20]. Our research has incorporated a genome-based phylogenetic analysis using the Genome Taxonomy Database (GTDB) to enhance the effectiveness of SPECIEs [21]. By exploring the phylogenetic distribution of electrotrophic acetogens in the Firmicutes phylum and related lineages, we aimed to identify the strains with significant potential for MES applications. This approach allowed for a more precise understanding of these organisms’ phylogenetic relationships, aiding in the discovery of their diversity and potential. In addition to the phylogenetic analysis, we conducted experiments to validate the electrotrophic capabilities of the selected strains under electrode-oxidizing conditions. These experiments were pivotal in confirming the electrotrophic nature of the strains and exploring their phylogenetic diversity and metabolic processes. The results from our study have highlighted the potential of SPECIEs as a valuable tool for the isolation of environmental microorganisms, particularly electrotrophic acetogens. While the current iteration of SPECIEs shows promise, further refinements could make significant contributions to the field of microbial isolation techniques. Importantly, the advancement of SPECIEs could play a crucial role in expanding the selection of the biocatalysts available for MES. By broadening the range of electrotrophic acetogens that can be effectively isolated and utilized, SPECIEs may substantially enhance the versatility and efficiency of MES applications in the future.

## 2. Materials and Methods

### 2.1. SPECIEs: A Novel Approach for Electrotrophic Acetogen Isolation

**Figure 1:**
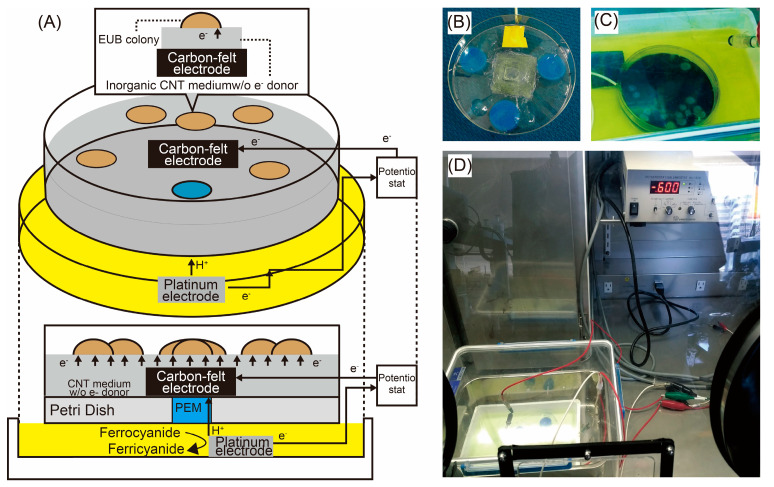
The conceptual and actual operation of SPECIEs: (**A**) is the conceptual design of SPECIEs, (**B**) is the Petri dish before pouring the medium, with the proton exchange membrane set in the center; (**C**) is the actual colony forming; (**D**) is a general view of the culture apparatus, including potentiostat. The culture was operated in an anaerobic box.

The imperative requirement for advancing microbial electrosynthesis (MES) technologies lies in the effective isolation and utilization of diverse electrotrophic acetogens. Traditional methods for isolating these bacteria from environmental samples have been limited in scope and efficiency. Recognizing this gap, we developed SPECIEs (Solid-Phase Electrochemical Isolation Equipment), an innovative solid-phase electrochemical culture system. By ingeniously integrating multi-walled carbon nanotubes (CNTs) into the conductive medium, SPECIEs leverages the medium itself as an electrode. This design enables electrotrophic acetogens to directly acquire electrons from their environment, promoting their growth under applied electric potential (Figure 1A).

Run Nomenclature: Throughout this section, we will label the experimental setups using shorthand notation. The setups with voltage applied will be labeled as “Run-XE”, where “X” is the run number and “E” stands for “Electrified”. Correspondingly, the setups without voltage will be labeled as “Run-XN”, where “N” stands for “Negative-control”. For instance, “Run-1E” signifies the first run in which voltage is applied, while “Run-1N” refers to the corresponding setup without voltage as a control.

Prototype SPECIEs (Run-1E and Run-1N): The prototype SPECIEs setup for Run-1 was constructed as follows: A square hole of approximately 3 cm was cut into the center of the bottom of a plastic Petri dish. An ion exchange membrane (Nafion 117) was used to cover the hole using Ultra Multi-Purpose SU adhesive (Konishi Co., Ltd., Tokyo, Japan) (Figure 1B). A carbon felt electrode of 10 cm^2^ was attached to the ion exchange membrane using Nafion 117 dispersion liquid (Wako, Japan). For the preparation of the solid medium, multi-walled carbon nanotubes (CNTs, final concentration 1 wt%, Meijo Nano Carbon Co., Ltd., Aichi, Japan) and gellan gum (final concentration 1.5%) were incorporated into the liquid medium composition (composition of liquid medium is detailed in Section 2.6). The medium was autoclaved, and 20 mL was dispensed into each Petri dish, ensuring that the carbon felt electrode was fully submerged. The central protonic membrane region of the Petri dish was soaked in a 50-mM potassium ferrocyanide solution (Figure 1C). A platinum black electrode served as the counter electrode, consistent with the protocol outlined in Section 2.6. After solidification, the Petri dishes were sterilized with UV irradiation for one day. A Ag/AgCl_2_ electrode served as the reference electrode, and a potentiostat (HA-151, Hokuto Denko) was used to apply a standard redox potential of −600 mV to the anode. An inoculum derived from reservoir water from the Yabase oil field [22] and acclimated for an extended period with H_2_/CO_2_ was used as the source of the anaerobic microorganisms. A 100-μL drop of this inoculum was applied to the center of each Petri dish. One setup received an applied voltage (Run-1E), while the other served as a control (Run-1N). Both were incubated for one month at room temperature in an anaerobic box (BACTRON 300, Shell Lab, OR, USA) filled with N_2_ to maintain anaerobic conditions (Figure 1D).

Improvements and Modifications to SPECIEs: Run-2 and Run-3: To address the issues identified in the initial SPECIEs setup (discussed later in the Results Section), we prepared the following four new setups: Run-2E, Run-2N, Run-3E, and Run-3N. The medium was modified from that used in Run-1, as follows: gellan gum was omitted and the CNT concentration was adjusted to 0.1%. Additionally, 10% sodium silicate was incorporated. The modified medium was poured into Petri dishes, and hydrochloric acid was added dropwise to achieve a pH of 7, inducing gelation. The medium was sterilized via UV irradiation for one day. Both the reference electrode and the potentiostat remained unchanged from the initial setup. Run-2E was operated at a potential of −600 mV and incubated for one month in an anaerobic box, similar to Run-1. Its negative control, Run-2N, also lacked any applied voltage and was incubated in the same conditions. Run-3E and Run-3N followed a similar protocol, which featured a three-month incubation period in an anaerobic box, with a −400-mV potential applied to Run-3E.

### 2.2. Genomic DNA Extraction and PCR Conditions

Genomic DNA was extracted from SPECIEs colonies using a MonoFas Bacterial Genomic Kit VII (GLC science, Tokyo, Japan) according to the manufacturer’s instructions, and the concentration and purity were measured using a NanoDrop ND-1000 spectrophotometer (Thermo Fisher Scientific, Waltham, MA, USA). Using the genomic DNA as a template, the V3–V4 variable regions of the 16S rRNA genes were amplified using a KAPA HiFi HotStart ReadyMix kit (Kapa Biosystems, Wilmington, MA, USA) with the following bacterial domain-specific primers: 341F (5′-TCGTCGGCAGCGTCAGATGTGTATAAGAGACAGCCTACGGGNGGCWGCAG-3′) and 805R (5′-GTCTCGTGGGCTCGGAGATGTGTATAAGAGACAGGACTACHVGGGTATCTAATCC-3′) [23]. For the amplification of the 16S rRNA genes, the samples were subjected to initial denaturation at 94 °C for 2 min, followed by 25 cycles of denaturation at 95 °C for 10 s, annealing at 55 °C for 30 s, and extension at 72 °C for 30 s, with a final extension at 72 °C for 5 min.

### 2.3. Sequencing Library Preparation, Sequencing, and Bioinformatics Analysis

The lengths of the PCR products were confirmed via 1.0% agarose gel electrophoresis, and the resulting amplicons (approximately 450 bp in length) were purified using Agencourt^®^ AMPure XP (Beckman Coulter, Brea, CA, USA) magnetic beads. The purified amplicons were then indexed through tailed PCR utilizing a Nextera XT Index Kit v2 Set A (Illumina, San Diego, CA, USA), followed by another round of purification according to the aforementioned process. The concentration of the tailed PCR amplicons was measured using a Quantus Fluorometer and the Quanti Fluor™ dsDNA System (Promega, Madison, WI, USA). After further adjustments of the concentration, the libraries were prepared. The sequencing of the libraries was performed using a MiSeq sequencer (Illumina) along with a MiSeq Reagent Kit v3 (Illumina).

### 2.4. Phylogenetic Tree of Electrotrophic Acetogens and Their Characterization

We employed phyloT [24], a service for creating GTDB-based phylogenetic trees, to visualize the extended lineage of *Firmicutes* (encompassing provisional categories of *Firmicutes* A–G) and closely related phyla, such as *Actinobacterota*, *Armatimonadota*, *Dormibacterota*, *Chloroflexota*, and *Eremiobacteria*. This grouping essentially represents the majority of *Terrabacteria* [25]. We generated an order-level phylogenetic tree using phyloT and then visualized it using iTOL [26], an online tree-rendering service. On iTOL, the order-level phylogenetic tree was clustered and labeled at the phylum level, recognizing that, while we refer to Firmicutes A–G as phyla for convenience, our use of GTDB includes several unvalidated taxonomic ranks.

To estimate the lineages capable of acetogenesis through the WLP, we utilized Annotree [27], a gene search service aligned with GTDB phylogenetic trees. Our screening focused on genomes possessing the CODH/ACS gene modules (KEGG IDs: K00192, K00193, K00194, and K00197) and the phosphotransacetylase gene (K00625). Additionally, the presence of bifurcating hydrogenase, crucial for the input of hydrogen-derived electrons into WLP, was determined using a combination of Annotree and HydDB [28]. Annotree was used to screen for bifurcating hydrogenase (K17997), and HydDB (a hydrogenase database) was employed to identify the genomes with [FeFe] Group A3 hydrogenases (representative of bifurcating hydrogenases).

While we could systematically investigate the distribution of hydrogenotrophic acetogens through genomic screenings, the same approach was not feasible for electrotrophic acetogens, due to the lack of identified genes related to electrotrophy. Consequently, we supplemented our analysis with a comprehensive literature review to identify the known electrotrophic acetogens and incorporated this information into our phylogenetic tree. This additional step ensured that our phylogenetic analysis accurately reflected the distribution of both hydrogenotrophic and electrotrophic acetogens, providing a more holistic view of the potential acetogenic bacteria within these phyla.

### 2.5. Selection and Culture of Electrotrophic Acetogen Candidates

Our study examined the electrotrophic potential of the following three bacterial strains: *Syntrophomonas erecta* JCM 13344, *Halanaerobacter jenidensis* JCM 16696, and *Desulfotomaculum defluvii* JCM 14036. These strains were chosen for their phylogenetic positioning within the *Firmicutes* phylum, with JCM 13344 and JCM 14036 belonging to *Firmicutes* B and JCM 16696 belonging to *Firmicutes* F. While JCM 13344, lacking the CODH/ACS gene module, served as a negative control, JCM 16696 and JCM 14036 showed potential as hydrogenotrophic acetogens. Our investigation aimed to uncover the unexplored electrotrophic capabilities of these strains, contributing to a broader understanding of the metabolic diversity within the Firmicutes lineage.

The bacteria were cultured at room temperature in a medium composed of the following: NH_4_Cl (1 g/L), KCl (0.1 g/L), MgSO_4_·7H_2_O (0.2 g/L), NaCl (0.8 g/L), KH_2_PO_4_ (0.1 g/L), CaCl_2_·2H_2_O (0.02 g/L), trace element solution (100 μL), Wolfe’s vitamin solution (100 μL), NaHCO_3_ (2 g/L), and cysteine (0.3 g/L). The medium was dispensed into 20-mL vial bottles and degassed to create anaerobic conditions. Post-degassing, the bottles were filled with CO_2_ and sterilized via autoclaving. Following sterilization, 10 mM of fructose and bacteria were added, and the bottles were pre-cultured for one month. The cultures were used for the cultivation of the electrode-oxidation conditions shown in the next section.

### 2.6. Electrochemical Culture of Selected Bacteria

H-type glass reactors were selected for culture under electrode-oxidation conditions due to their autoclavability and suitability for cultivating pure bacterial strains while minimizing the risk of contamination. In these reactors, the anode and cathode compartments were separated with a proton exchange membrane (Nafion 117, Sigma-Aldrich, St. Louis, MO, USA). Holes were drilled into the lid of each compartment to accommodate the electrodes. Then, a carbon felt electrode (10 cm^2^, Toyo Tanso Co. Ltd., Tokyo, Japan) and a reference electrode (Ag/AgCl_2_ electrode, RE-1C, BAS, Tokyo, Japan) at +197 mV were inserted on the cathode side, while a platinum black electrode was inserted on the anode side. A potentiostat (HA-151B, Hokuto Denko, Tokyo, Japan) was employed to apply a potential of 600 mV. Usually, such voltage-applied incubators are used in microbial electrolytic cells (MEC), but they are also frequently used to investigate the bioactivity of strains in pure culture under voltage-controlled conditions. The construction of the pure-culture MES was based on the report of Bajracharyan et al. [29]. The medium for the electrode cultures was identical to the pre-culture medium, among other elements, but omitted fructose. Additional agents such as resazurin and EDTA were added to the cathode side of the medium, while potassium ferrocyanide served as an electron source on the anode side. Bacteria pre-cultured for one month at room temperature were introduced into the cathode compartment. The system was operated for one week, and electrode samples were collected both immediately following the bacterial inoculation and at the end of the operation period.

### 2.7. Lowry Method for Protein Quantitation 

After accurately pipetting 0.2 mL of the sample into a clean test tube, 1 mL of copper solution was added and it was thoroughly mixed, followed by a 10-min incubation period. For the reagent blank, ion-exchanged water was used in place of the sample solution. After the 10-min incubation, 0.1 mL of 1 N phenol reagent was added and the solution was mixed thoroughly, and then the sample was incubated at room temperature for 30 min. Subsequently, the protein content was determined using the Lowry method by measuring the absorbance at a wavelength of 750 nm using a spectrophotometer. Each sample was measured three times. Additionally, the bacterial density was calculated based on the volume of each sample.

## 3. Results and Discussion

### 3.1. Electrochemical Cultivation of Potential Acetogens on Solid Medium

The SPECIEs prototype showcased in Run-1 was operated for 4 weeks in an anaerobic box. The operating conditions are described in Table 1. Multiple colonies were identified at the end of the Run-1E cultivation, among which the largest was found to be a fungal colony upon microscopic observation (data not shown). SPECIEs cultivates under potential-controlled conditions with a potentiostat. In Run-1E, the resazurin added to the medium did not change color, suggesting that the medium was maintained in a reduced state, confirming that there was no oxygen present in the anaerobic box. However, the growth of fungi was still observed. While fungi are generally known to be aerobic organisms, some strains have been observed to grow under strictly anaerobic conditions [30], therefore, we speculated that this was the case with the growth observed on the SPECIEs Run-1 medium. The gelling agent used in Run-1 was gellan gum, which itself is organic, suggesting that the conditions were not strictly inorganic and that the fungus likely proliferated using the gellan gum as a substrate. Additionally, the cultivation was carried out in a sealed container, thereby ruling out the possibility that contamination from inside of the anaerobic box caused fungal colony formation (Figure 1C). 

SPECIEs is designed to isolate colonies of acetogenic bacteria under electrode-oxidation conditions, therefore, it is necessary to avoid the formation of fungal colonies. As it was speculated that the use of gellan gum as a gelling agent was a factor in the proliferation of the fungus, sodium silicate, adjusted to pH 7.0, was used as a gelling agent under strictly inorganic conditions, and other medium components were added to the medium for Run-2. In Run-2E, a potential of −600 mV was applied, and cultivation was carried out for 1 month, as in Run-1; however, no detectable colonies were observed. In Run-3, more moderate conditions were used, with the applied voltage set to −400 mV, and the cultivation period was extended to 3 months. Under these cultivation conditions, the formation of particles thought to be mini-sized colonies were confirmed on the surface of the medium after 3 months (Run-3E).

**Figure 2:**
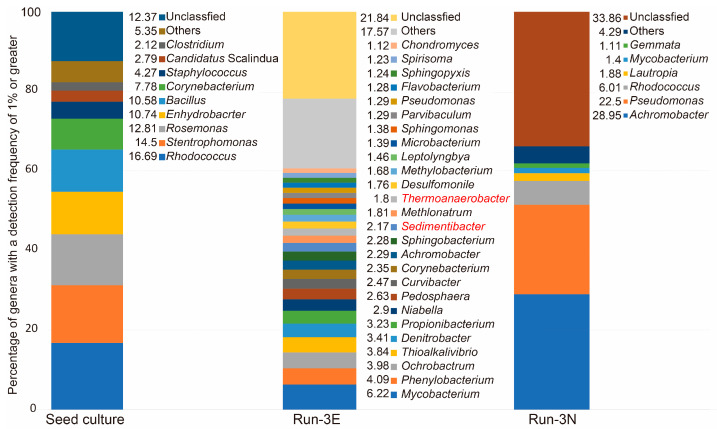
Comparative analysis of the microbial community composition, including formed microcolonies. The source culture represents a sample from oil reservoir water stored at 55 °C from the Yabase oil field. Run-3E shows a sample cultured under −400-mV (vs. Ag/AgCl_2_) conditions using SPECIEs. Run-3N represents the microbial community formed without the application of voltage in SPECIEs. Genera with a detection frequency exceeding 1% were selected from each microbial community analysis and plotted in a stacked graph. The numbers on the left side of the legend indicate the percentage represented by each genus. The genera highlighted in red indicate the presence of genomes reported to have CODH/ACS and phosphotransacetylase activities.

In order to identify the microbial communities comprising the microcolonies that were successfully generated, we acquired colonies from the solid medium surface of Run-3E and 3N. We also analyzed the microbial community of the seed source (reservoir water from the Yabase oil field, where the headspace was replaced with H_2_/CO_2_ (80%/20%), which was stored at 55 °C) to eliminate the possibility of carryover from the seed source. The results, shown in Figure 2, demonstrate that the microbial communities of the three systems differed extensively. The stacking of genera with a detection frequency of ≥1% in a graph revealed almost no genera common to the three systems, suggesting that the microbial communities detected in Run-3E and 3N were not likely to be the result of carryover from the seed source but instead formed on the SPECIEs medium surface. This implies that species that may have been present at less than 1% in the seed culture became predominant on the SPECIEs plates. Importantly, the microbial community of Run-3E with voltage application was extremely diverse. Compared to the simplicity of the microbial community of Run-3N, it can be said that we successfully cultured a variety of bacteria. Furthermore, two genera of bacteria belonging to phylum Firmicutes A (Thermoanaerobacter and Sedimentibacter) were found among the detected species. Some reports have suggested that these genera include strains capable of utilizing electrons [31,32]. Both of the genera have been reported to have genomes equipped with the CODH/ACS gene module and phosphotransacetylase genes (GCF_000763575, GCA_002399975), indicating a high likelihood of being acetogens.

The specific detection of these strains in Run-3E demonstrates the feasibility of culturing electrotrophic acetogens using the SPECIEs method. However, it is also true that other species of bacteria were detected, despite the inorganic conditions. The most frequently sequenced genus, Mycobacterium, which accounted for 6.22% of the isolates, is known to include autotrophic bacteria that utilize hydrogen. Therefore, if these organisms can utilize the electrode as if it were hydrogen, this could explain the colony formation [33]. On the other hand, to date, there have been no reports that Phenylobacterium, which represented 4.09% of the isolated genera, is autotrophic, suggesting that the colony formation involves a different pathway. Additionally, the detection of genera typically considered aerobic in Run-3E may indicate that the anaerobic chamber did not maintain a completely anaerobic atmosphere. However, the addition of resazurin remained in a fully reduced state and did not change color. This suggests that an environment conducive to the growth of strictly anaerobic bacteria, such as acetogens, was established, as evidenced by the detection of acetogenic colonies. The presence of aerobic bacteria in the microbial community analysis hints at the possibility that these organisms possess some metabolic pathways that enable them to proliferate in anaerobic conditions. However, this remains speculative, and further exploration, such as predicting the metabolic functions of the SPECIEs colony groups through metatranscriptomics, is necessary. Similarly, ensuring that the oxygen concentration remains below detectable limits is equally as important.

Many of the other isolated bacterial species were also from heterotrophic genera, suggesting the presence of colonies utilizing organic matter fixed by autotrophic bacteria such as Mycobacterium, Thermoanaerobacter, and Sedimentibacter. In other words, although SPECIEs can be used to directly form and culture colonies of electron-utilizing acetogens from environmental samples on a solid medium, it can also be said that it is not strictly selective for acetogens. To improve the specificity of SPECIEs, it will be necessary to develop strategies to eliminate the competing autotrophic bacteria and heterotrophic bacteria. By simply adjusting the size of the culture device and the colony density on the plate to increase the distance between colonies, it would be possible to limit the supply of organic matter to the heterotrophic bacteria.

On the other hand, it is also intriguing that, despite employing cultivation conditions involving minimal organic content (only vitamins) and using sodium silicate as a gelling agent, a diverse colony was formed in Run-3E using the SPECIEs device. Although the redox potential is known to significantly impact microbial cultivation [34], there are no known instances of this control being examined on a solid medium. The SPECIEs device could potentially uncover microbiomes that have not been cultured in the past. In other words, SPECIEs is a technology that links two contrasting propositions, improving the specificity for acetogens and enhancing the potential of making them universally cultivable. Of course, these two propositions do not necessarily need to be satisfied simultaneously, and elucidating the two paths to satisfy each represents development challenges for the future.

In SPECIEs Run-3E, the use of sodium silicate as a gelling agent under strictly inorganic conditions for extended voltage application led to the acquisition of a diverse array of colonies, including those of Firmicutes A. This methodology offers a substantial advantage by directly isolating and culturing electrotrophic organisms from environmental samples, using electrotrophy as a selection pressure. The traditional methods for studying electrotrophic organisms frequently suffer from biases—most notably at the enrichment stage—where only those microorganisms that can adapt to specific environmental conditions are allowed to proliferate, compromising the original diversity of the microbial community. While SPECIEs is not entirely free from biases relating to colony-forming ability, it bypasses the issue of enrichment bias, thereby increasing the likelihood of isolating previously uncultured lineages of electrotrophic organisms.

For advancing MES technologies, SPECIEs offers a promising avenue for isolating acetogens, particularly from Firmicutes. In this context, a set potential of −400 mV serves as a carefully chosen threshold, balancing the need to avoid abiotic hydrogen production while also providing a sufficient electrochemical driving force for ferredoxin reduction in the WLP. However, it should be noted that this potential may not fully negate the influence of certain acetogens, such as those from the genus Sporomusa. These organisms are suspected of secreting extracellular substances, possibly hydrogenases, that could enable abiotic hydrogen production, even at −300 mV [35]. Therefore, while −400 mV is effective for avoiding most abiotic hydrogen production, its adequacy in preventing hydrogen production from these specific acetogens remains a challenge. Given SPECIEs’ solid-medium design, the diffusion of these extracellular secretions is expected to have a less pronounced impact on the microbial community than that observed in liquid cultures. 

Going forward, identifying substances that are capable of serving as effective electron shuttles between the electrode and cellular electron entry points remains a priority. Currently, no known mediators are available for this role in electrotrophy, but any chosen mediator must have a redox potential lower than that of ferredoxin to drive WLP effectively. Quinones [36] are not suitable for this purpose, while methyl viologen, previously used as an electron donor in ferredoxin-mediated reactions within anaerobic Firmicutes (e.g., Clostridium), presents itself as a potential candidate [37]. The challenge ahead lies in optimizing SPECIEs both in terms of applied voltage and mediator selection in order to make it a robust tool for the selective cultivation of electrotrophic acetogens. This is particularly critical for advancing electrode-oxidation-driven WLP, a promising yet challenging frontier in MES research.

### 3.2. Phylogenetic Analysis of Acetogens: Elucidating Latent Electrotrophic Traits within Firmicutes Sensu Lato

**Figure 3:**
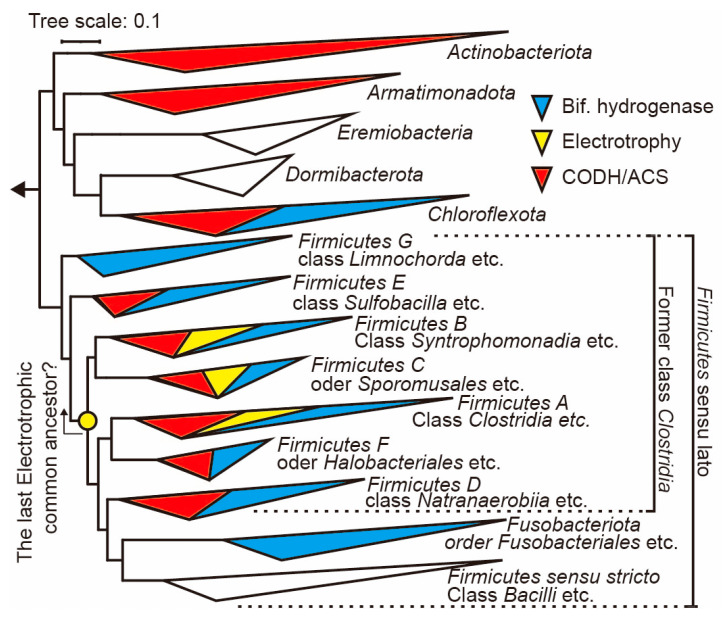
Phylogenetic tree *Firmicutes* sensu lato and nearby phylogenetic groups (i.e., *Terrabacteria*) by GTDB. Clusters are indicated at the phylum level, and clusters containing strains with CODH/ACS genes, electrotrophic-utilizing activity, and bifurcation hydrogenase activity are indicated in red, yellow, and blue, respectively.

Figure 3 illustrates the GTDB-based phylogenetic tree constructed in the present study. This tree clustered groups of Firmicutes sensu lato and their closely related taxa (Actinobacteriota, Chlorofexota, Armatimonadota, Dormibacterota, and Eremiobacterota), as reported in previous studies, at the phylum level. The triangular areas of the clusters represent the diversity within each phylum. We investigated the genomes within each cluster using Annotree, screening for clusters that include genomes with the CODH/ACS gene module (central enzymes of the Wood–Ljungdahl pathway) and the terminal phosphotransacetylase enzymes. Furthermore, we narrowed down to genomes that also possess the Fe-Fe bifurcating hydrogenase, essential for supplying NADH and ferredoxin to drive the WLP, using both Annotree and HydDB. The genomes harboring these enzymes have genes responsible for the initiation (H_2_ oxidation), midpoint (acetyl-CoA synthesis), and terminal point (acetate synthesis) of the WLP. In this study, bacteria with these characteristics were evaluated as potential hydrogenotrophic acetogens. The clusters containing genomes with the CODH/ACS gene module were marked in red. Those with bifurcating hydrogenase were indicated in blue, and the corresponding assembly and protein information are presented in Table 2. Moreover, information on the strains demonstrating electrotrophic activity was collected through a literature review, with reported examples in Firmicutes A, B, and C listed in Table 3 and indicated in yellow in Figure 3. In Table 2, a ‘+’ sign is used to denote the strains or genomes where hydrogenotrophy or electrotrophy was suggested at the strain or genome level. It should be noted that the absence of reports in a particular family does not necessarily mean a lack of activity within that family; hence, ‘N.R.’ (not reported) is used to indicate.

As a result of the phylogenetic and physiological activity analysis in this study, it was determined that potential acetogens, as defined herein, are distributed within Chloroflexota and Firmicutes sensu lato. The potential acetogens detected in Chloroflexota were found in a metagenome derived from wastewater and are presumed to belong to the class Dehaloccocoidia (GCA_002436065). Including this example, there have been two reported instances of genomes coding for Fe-Fe bifurcating hydrogenase in Chloroflexota, suggesting the possibility of functional autotrophic metabolism starting from hydrogen in the Dehaloccocoidia class metagenome, which is intriguing. However, aside from this single instance, all of the other potential acetogens identifiable within Terrabacteria were found to be within Firmicutes sensu lato. The genomes within the provisional GTDB ranks of Firmicutes A–F were found to code for the CODH/ACS gene module, phosphotransacetylase, and bifurcating hydrogenase. Interestingly, no genomes coding for the CODH/ACS gene module and bifurcating hydrogenase were detected in Firmicutes sensu stricto, a clade centered around the class Bacilli, and the only clade within Firmicutes sensu lato that has adapted to aerobic environments. This suggests the possibility that the WLP was discarded during the adaptation process. Firmicutes A–F, despite various opinions, have traditionally been classified within the class Clostridia, according to the 16S rRNA-gene-based taxonomic system (NCBI taxonomy), as indicated in Figure 3 as ‘former Clostridia’ [38]. For example, the genus Sporomusa, belonging to Firmicutes C, has been classified in the phylum Negativicutes, according to NCBI taxonomy; however, there has been persistent debate about this being a misclassification, and it is argued that they actually belong to the class Clostridia. Including these, the class Clostridia in NCBI taxonomy is thought to encompass acetogens [39]. The GTDB-based taxonomic classification reveals that the diversity of acetogens is not confined to a class but is distributed at the phylum level.

Regardless of the aforementioned issue, the clusters color-coded in red, blue, and yellow are located within the phylum Firmicutes sensu lato. Hydrogenotrophy and electrotrophy serve as energy inputs into the cells, and, in acetogens, this energy is utilized as reducing power in the Wood–Ljungdahl pathway (WLP), which is widely regarded as one of the oldest carbon and energy fixation routes. Recent genome-based phylogenetic analyses suggest that the direct descendants of the last universal common ancestor (LUCA) belong to the class Clostridia (i.e., Firmicutes sensu lato in GTDB) and to methanogens [40]. This implies that the traits of hydrogenotrophy or electrotrophy, acting as sources of reducing power for the WLP, were likely acquired by the ancestor of both groups and then transmitted vertically [41]. However, it is important to note that the electrotrophy of acetogens has not yet been definitively confirmed. This uncertainty stems from the possibility that abiotic hydrogen may be produced on metal surfaces, such as electrodes or iron, and this hydrogen could be utilized instead of direct electron uptake [42]. Therefore, whether the strains listed as electrotrophic in Table 1 truly utilize electrons directly remains a subject of discussion. Nevertheless, methanogens, which are direct descendants of LUCA, are known to be not only hydrogenotrophic but also strictly electrotrophic [43]. If electrotrophy is the metabolism of LUCA, it stands to reason that acetogens would also exhibit electrotrophy, similar to methanogens. Moreover, one clear conclusion that can be drawn from Figure 1 is that hydrogenotrophic and electrotrophic acetogens are widely distributed within the Firmicutes sensu lato. If these traits were transmitted vertically from ancestral bacteria, this would suggest that all bacteria within the Firmicutes sensu lato could be acetogens (i.e., WLP users), except for those that have lost this capability through evolutionary processes. In essence, the provision of some form of autotrophic reducing power is essential for driving the WLP. Therefore, we can hypothesize that all of the members of the Firmicutes sensu lato are potentially hydrogenotrophic and/or electrotrophic acetogens.

### 3.3. Evaluating Latent Electrotrophic Potential in Selected Firmicutes Strains: Uncovering New MES Candidates 

In pursuit of broadening our understanding of the electrotrophic capabilities within the Firmicutes phyla, we conducted a study focusing on the strains traditionally linked to hydrogenotrophy but potentially harboring unexplored electrotrophic traits. Our methodology involved the selection of specific strains from Firmicutes B and F, namely D. defluvii JCM 14036 and H. jeridensis JCM 16696. These selections were informed by genome-based analyses, indicating the presence of metabolic genes such as CODH/ACS complexes and bifurcating hydrogenases in their close relatives, suggesting their latent hydrogenotrophic acetogenic potential. Additionally, the electrotrophic traits of these strains were uncharted, offering a unique opportunity to identify and characterize novel electrotrophic acetogens. Our approach aimed to challenge the traditional understanding of these bacteria and expand the known spectrum of electrotrophic acetogens by cultivating these strains under electrode-oxidizing conditions.

Among the strains tested under such electrode-oxidizing conditions, H. jeridensis JCM 16696, belonging to Firmicutes F, was the only one to exhibit an increase in protein mass. This growth was observed on the electrode surface and in the planktonic cells under the conditions of electrode oxidation and inorganic nutrient provision. The growth pattern, characterized by a gradual increase over a one-week period, differed significantly from the other two strains, S. erecta JCM 13344 (e.g., negative control) and D. defluvii JCM 14036, both from Firmicutes B, which showed a decrease in protein mass under similar conditions. The observed growth of H. jeridensis JCM 16696 highlights the potential for electrotrophic activity within Firmicutes F, marking a significant finding, as it suggests the possibility of electrotrophic capabilities in this phylum for the first time. This result contributes to the expansion of our understanding of the electrotrophic potential within the Firmicutes phyla, indicating a potentially wider distribution of these capabilities than previously understood.

To further elucidate our findings, we suggest that, within the class of hydrogenotrophic Firmicutes, which also utilize the Wood–Ljungdahl pathway (WLP), there may exist bacteria that employ electrons directly from electrodes as a source of reductive power, rather than relying solely on hydrogen. This distinction is crucial, because hydrogen and electrons are incorporated into metabolic pathways in different ways, as follows: In WLP, hydrogen is used as a reductive agent via electron-bifurcating hydrogenase to reduce ferredoxin (Fd) and NAD+ [44]. Electrotrophy, on the other hand, directly utilizes the electrons from electrodes, thereby bypassing the need for hydrogen as an intermediary electron donor. If electrotrophs indeed use electrodes for direct electron uptake, intracellular ferredoxin reduction (Fd + 2e^−^ → Fd2^−^) would occur independently of electron-bifurcating hydrogenase. Notably, the redox potential of −600 mV (vs SHE) is compatible with the midpoint potential of ferredoxin, typically around −400 mV, providing a sufficient driving force for electron transfer. This is in contrast to NADH, whose midpoint potential is typically too high (around −320 mV, depending on the conditions [45]) to be an effective electron donor for ferredoxin reduction in WLP. If NADH were the primary entry point for electrons, its high redox potential would impede efficient ferredoxin reduction, thus affecting the functionality of WLP. Instead, specialized proteins such as polyferredoxins [46] and multi-heme cytochromes [47] offer a more direct and electrochemically favorable pathway from the electrode to ferredoxin, circumventing the need for a less suitable electron carrier like NADH. However, it is important to note that, while multi-heme cytochromes have been implicated in the electrotrophy of other lineages, such as *Desulfovibrio* and *Methanosarcina*, there are no reported instances of multi-heme cytochromes being exposed on the cell membrane in bacteria from the Firmicutes A-F phyla.

These considerations regarding direct electron uptake offer not only a biochemical pathway, but also insights into the evolutionary biology of metabolism. The direct channeling of electrons into ferredoxin might represent an ancient metabolic strategy, possibly dating back to the last universal common ancestor (LUCA), before the development of electron-bifurcating hydrogenases. It is believed that the evolution of electron-bifurcating hydrogenase occurred independently in acetogens and methanogens after they diverged from LUCA [48]. This suggests that the current mechanisms of hydrogen utilization in bacteria utilizing the Wood–Ljungdahl pathway (WLP), through electron-bifurcating hydrogenase, may be relatively newer adaptations. Hence, electrotrophy might be an older metabolic pathway that existed before these adaptations. The emergence of electron-bifurcating hydrogenase likely introduced more complexity and flexibility in driving the WLP. The universality of electrotrophy across all of the lineages within the Firmicutes phyla, including those traditionally associated with hydrogenotrophy, is still a matter of investigation. However, our findings, particularly with electrotrophy from Firmicutes F, suggest that electrotrophy might be a latent feature within these groups. This potentially indicates that electrotrophy could be inherent in many bacteria within these phyla, except possibly in those strains that may have lost this capability through evolutionary processes.

Our culture data, particularly from *H. jeridensis*, support the idea that electrotrophy may be a latent ability within Firmicutes. Given the limitations of traditional culture-based methods, electrode-oxidizing conditions offer a targeted strategy for future isolations. In the following section, we will explore how electrochemical cultivation techniques can further advance this area of research.

### 3.4. Development Potential and Limitations of SPECIEs and Future Challenges

The current challenge with SPECIEs lies in its lack of specificity. The application of voltage promoted colony formation in a wide range of bacterial groups, leading to the detection of diverse bacterial colonies on the SPECIEs plates, despite cultivation under autotrophic conditions. This poses significant noise in the isolation of acetogens and contradicts SPECIEs’ primary objective of specifically and directly acquiring electrotrophic acetogens from the environment for MES applications. If the aim is simply to obtain bacteria with high fermentation productivity, chemostat enrichment cultivation using liquid media in MES might be more suitable, as several studies have already shown [49]. On the other hand, the scarcity of acetogen biocatalysts for MES is a problem. The genetic resource limitations directly impact the range of producible substances. Despite *Sporomusa*’s high compatibility with MES, the inability to genetically modify these strains has limited MES’s primary products to acetate for over a decade since its first report [50]. Advancement in MES likely necessitates a shift towards a synthetic biology paradigm through genetic modification, and a diverse genetic resource of acetogens is indispensable for this transition. The detection of colonies considered acetogens from SPECIEs’ microbial consortia indicates that the device functions at least as a non-specific isolation and cultivation apparatus for acetogens. If specificity improvements are achieved, SPECIEs has the potential to strongly assist in supplying genetic resources for MES directly from environmental samples.

**Table 2:** List of the distribution of hydrogenotrophic and electrotrophic bacteria within several phyla within *Terrabacteria*. Each activity is indicated by + if it was recognized, and not reported (N.R.) if there are no reported cases.

			Activity						
	Phylum	Class	CODH/ACS Genes	pta Gene	Electron Utilizing	Bifurcating Hydrogenase Gene	Species Name/MAG Source	Assembly/Protein Accession	Reference of E-Utilizing Activity
	*Actinobacteriota*	*Coriobacteriia*	+	+	N.R.	-	Rumen	GCA_900314665	
	*Aromatimonadota*	*Abditibacteria*	+	+	N.R.	-	groundwater filtered through a 3.0 um filter	GCA_001872605	
	*Eremiobacteria*	-	-	-	N.R.	-	-	-	
	*Dormibacterota*	-	-	-	N.R.	-	-	-	
	*Chloroflexota*	*Dehaloccocoidia*	+	+	N.R.	+	wastewater	GCA_002436065	
Firmicutes sensu lato	*Firmicutes* G	UBA4882	-	-	N.R.	+	*Hydrogenispora ethanolica*	GCF_004340685	
	*Firmicutes* E	DTU015	+	+	N.R.	+	Hydrothermal vent biofilm	GCA_002291985	
	*Firmicutes* B	*Moorellia*	+	+	+	+	*Moorella thermoacetica*	GCF_001267405/WP_011393219	Nevin et al., 2011 [13]
	*Firmicutes* C	*Negativicutes*	+	+	+	+	*Sporomusa ovata*	GCF_000445445/WP_021167212	Nevin et al., 2011 [13]
	*Firmicutes* A	*Clostridia*	+	+	+	+	*Clostridium ljungdahli*	GCF_000143685/WP_013238135	Im et al., 2022 [51]
	*Firmicutes* F	*Halanaerobiia*	+	+	N.R.	+	*Acetohalanaerobium arabaticum*	GCF_000144695/WP_013277223	
	*Firmicutes* D	*Dethiobacteria*	+	+	N.R.	+	*Hydrothermal vent biofilm*	GCA_002292015	
	*Fusobacteriota*	*Fusobacteriia*	-	-	N.R.	+	Fusobacterium varium	WP_005950651	
	*Firmicutes* sensu stricto	-	-	-	N.R.	-	-	-	

**Table 3:** Increase in protein content with incubation under electrode-oxidation conditions. The strains used were *Syntriphomonas erecta* JCM 13344, *Halanaerobacter jenidensis* JCM 16696, and *Desulfotomaculum defulvii* JCM 14036.

Strain Name	Sampling Conditions	Protein Concentration (mg-BSA/mL) [A]	Total Volume of Sampling Locations (mL) [B]	Total Protein Weight at Sampling Location (mg) [A × B]	Net Protein Increase (mg)	Potential for Growth under Electrode-Oxidation Conditions
*Syntriphomonas* *erecta* JCM 13344	Planktonic cells post-cultivation	0.272 ± 0.079	200	68.733	−14.400	Negative
	Planktonic cells immediately after cell addition	0.344 ± 0.065	200	54.333		
	Electrode post-cultivation	0.278 ± 0.055	10	2.780	0.66	Negative
	Protein weight of the electrode immediately after cell addition (calculated value)			0.344 × 10 = 3.44		
*Halanaerobacter jenidensis* JCM 16696	Planktonic cells post-cultivation	0.692 ± 0.091	200	138.400	47.067	Positive
	Planktonic cells immediately after cell addition	0.457 ± 0.129	200	91.333		
	Electrode post-cultivation	1.227 ± 0.157	10	12.270	7.700	Positive
	Protein weight of the electrode immediately after cell addition (calculated value)			0.457 × 10 = 4.57		
*Desulfotomaculum* *defulvii* JCM 14036	Planktonic cells post-cultivation	0.395 ± 0.044	200	84.467	−5.533	Negative
	Planktonic cells immediately after cell addition	0.422 ± 0.052	200	78.933		
	Electrode post-cultivation	0.203 ± 0.086	10	2.027	−2.193	Negative
	Protein weight of the electrode immediately after cell addition (calculated value)			0.422 × 10 = 4.22		

The results of phylogenetic analysis and electrocultivation are crucial when discussing SPECIEs’ specificity. Electrochemical cultivation identified a strain in Firmicutes F suggesting electrotrophic capabilities. If electrotrophy in Firmicutes A, B, C, and F operates through a common mechanism, it implies that this trait could be an ancestral characteristic, as indicated by the yellow circles in the phylogenetic tree shown in Figure 3. This suggests the possibility of preserved electrotrophy in bacteria belonging to these phyla. This insight will be important in improving SPECIEs’ specificity, providing a key indicator of ‘what’ to cultivate. Enhancing specificity equates to narrowing diversity. Based on our findings, we can target Firmicutes A, B, C, and F for cultivation. Improving specificity, as mentioned in Section 3.1, might be achieved by adjusting the medium composition, device size, and seeding cell density, among others. These remain areas for future refinement.

## 4. Conclusions

Our research has successfully demonstrated the SPECIEs method’s capacity for cultivating a broad range of bacteria, including the identification of diverse microbial communities under inorganic conditions. This tool shows potential in advancing our understanding of microbial electrochemical systems, particularly in the isolation and cultivation of acetogens. However, this study has also revealed challenges in SPECIEs’ specificity, with a diverse array of bacterial colonies grown under electrotrophic conditions, indicating a need for further refinement in its application.

Additionally, our independent cultivation of *Halanaerobacter jeridensis* JCM 16696 under electrode-oxidizing conditions has revealed promising electrotrophic activity. This finding, in conjunction with our phylogenetic analysis, suggests a broader spectrum of electrotrophic capabilities within the Firmicutes phyla than previously recognized.

Together, these findings contribute significantly to the field of microbial electrosynthesis, highlighting the potential of electrotrophic bacteria in sustainable energy applications. Future improvements in the specificity of SPECIEs and further exploration of electrotrophic acetogens will enhance the efficiency and applicability of microbial electrochemical systems.

## Figures and Tables

**Table 1:** SPECIEs run conditions and evaluation of colony-formation ability. Colony formation was assessed, with + indicating confirmed colony formation, (+) indicating microcolonies, and − indicating no confirmed colony formation.

Run No.	Run-1E	Run-1N	Run-2E	Run-2N	Run-3E	Run-3N
Addition of voltage (mV)	−600	Not added	−600	Not added	−400	Not added
Final conc. of CNT	1%		0.10%		0.10%	
Gelling agent	Gellan gum		Sodium silicate (Adj. pH 7.0)		Sodium silicate (Adj. pH 7.0)	
Colony formation	+	+	−	−	+	−
Fungal colony formation	+	+	−	−	−	−
Cultivation period (weeks)	4		4		12	

## Data Availability

Data are contained within the article.
